# Suicide and the 2008 economic recession: Who is most at risk? Trends in suicide rates in England and Wales 2001–2011

**DOI:** 10.1016/j.socscimed.2014.07.024

**Published:** 2014-09

**Authors:** Caroline Coope, David Gunnell, William Hollingworth, Keith Hawton, Nav Kapur, Vanessa Fearn, Claudia Wells, Chris Metcalfe

**Affiliations:** a School of Social and Community Medicine, University of Bristol, Canynge Hall, 39 Whatley Road, Bristol BS8 2PS, UK; b Centre for Suicide Research, University of Oxford, Department of Psychiatry, Warneford Hospital, Headington, Oxford OX3 7JX, UK; c Centre for Suicide Prevention, Centre for Mental Health and Risk, Institute of Brain, Behaviour and Mental Health, The University of Manchester, Oxford Road, Manchester M13 9PL, UK; d Life Events and Population Sources Division, Office for National Statistics, Cardiff Road, Newport, Wales NP10 8XG, UK

**Keywords:** Suicide rates, suicide trends, Economic recession, England and Wales, Risk, Joinpoint regression

## Abstract

The negative impacts of previous economic recessions on suicide rates have largely been attributed to rapid rises in unemployment in the context of inadequate social and work protection programmes. We have investigated trends in indicators of the 2008 economic recession and trends in suicide rates in England and Wales in men and women of working age (16–64 years old) for the period 2001–2011, before, during and after the economic recession, our aim was to identify demographic groups whose suicide rates were most affected. We found no clear evidence of an association between trends in female suicide rates and indicators of economic recession. Evidence of a halt in the previous downward trend in suicide rates occurred for men aged 16–34 years in 2006 (95% CI Quarter 3 (Q3) 2004, Q3 2007 for 16–24 year olds & Q1 2005, Q4 2006 for 25–34 year olds), whilst suicide rates in 35–44 year old men reversed from a downward to upward trend in early 2010 (95% CI Q4 2008, Q2 2011). For the younger men (16–34 years) this change preceded the sharp increases in redundancy and unemployment rates of early 2008 and lagged behind rising trends in house repossessions and bankruptcy that began around 2003. An exception were the 35–44 year old men for whom a change in suicide rate trends from downwards to upwards coincided with peaks in redundancies, unemployment and rises in long-term unemployment. Suicide rates across the decade rose monotonically in men aged 45–64 years. Male suicide in the most-to-medium deprived areas showed evidence of decreasing rates across the decade, whilst in the least-deprived areas suicide rates were fairly static but remained much lower than those in the most-deprived areas. There were small post-recession increases in the proportion of suicides in men in higher management/professional, small employer/self-employed occupations and fulltime education. A halt in the downward trend in suicide rates amongst men aged 16–34 years, may have begun before the 2008 economic recession whilst for men aged 35–44 years old increased suicide rates mirrored recession related unemployment. This evidence suggests indicators of economic strain other than unemployment and redundancies, such as personal debt and house repossessions may contribute to increased suicide rates in younger-age men whilst for men aged 35–44 years old job loss and long-term unemployment is a key risk factor.

## Highlights

• Indicators showed economic hardship was increasing 5 years prior to the 2008 economic recession.
• The downward trends in younger male suicide rates (16–34 year olds) halted around 2006.
• Economic strain and unemployment may contribute to rises in suicide rates in men aged 35–44 years old.
• There was no clear evidence of an impact of the 2008 economic recession on suicide rates in females.

## 1 Introduction

March 2008 saw the beginning of an economic recession in the UK, marked by the first quarter of negative growth in gross domestic product (GDP) since 1991 (ONS, 2012) (see Appendix 1). Many other countries around the world were similarly affected by the global economic downturn, which was precipitated by the US banking crisis in 2007. In the UK the 2008 economic recession was preceded by rising levels of house repossessions and unmanageable household debt and followed by sharp rises in redundancies, unemployment, an increased proportion of part-time employed (under-employment) and reductions in wages. Many Governments introduced a series of austerity measures to restore economic growth, but these resulted in reductions in spending on public services and social protection programmes (Karanikolos et al., 2013).

It has long been recognised that periods of economic uncertainty are associated with rises in suicide (Durkheim, 1952, Morselli, 1882, Swinscow, 1951). Durkheim hypothesised that key societal forces such as social integration can be disrupted by factors related to economic downturn which consequently have an impact on suicide rates. Amongst a range of other stressors recessions lead to increases in debt, job losses, house repossession, strains on relationships and reductions in public spending, which in turn adversely affect mental health. A growing body of research has further clarified the association between economic recessions and rises in suicide rates. For example, the 1997–98 East Asian economic recession led to an estimated 10,400 excess suicides in Japan, Hong Kong and South Korea (Chang et al., 2009). Earlier recessions in Europe, the USA and Australia have also been associated with rises in suicide rates (Morrell et al., 1993, Stuckler et al., 2009, Stuckler et al., 2012).

It has been proposed that male suicide rates are more sensitive to unemployment levels than female suicide rates and that this is a cross-national phenomenon (Milner et al., 2012, Morrell et al., 1993). Increased unemployment has long been posited as a key driver of the rises in suicide that occur during economic downturns (Swinscow, 1951), although a theory of a simple association between rises in unemployment and suicide rates has been challenged by some authors (Fountoulakis et al., 2013). Studies from previous recessions indicate that the association between unemployment and suicide rates varies depending on the period, age, gender and geography of working age populations and that the impact differs depending on the robustness of a nation's social protection policies (Morrell et al., 1993, Stuckler et al., 2009). For example, in the recession of the 1990s when unemployment rates rose sharply both Finland and Sweden saw suicide rates drop, arguably as a result of their commitment to work programmes and social support during the crisis.

A series of studies reported increases in suicides following the 2008 economic recession in the UK, USA, Greece and much of Europe (Barr et al., 2012, Kentikelenis et al., 2011, Reeves et al., 2012). In their recent assessment of the global impact of the crisis Chang and colleagues (Chang et al., 2013) estimated that there were an additional 5000 suicides in 2009 compared to 2007 in the 54 countries they studied. Rises in unemployment accounted for some, but by no means all, of these rises in suicides (Barr et al., 2012, Chang et al., 2013). Evidence from previous crises shows there are adverse effects of anticipated job loss and job insecurity (Perlman and Bobak, 2009) and rises in suicide rates may be a response to early indicators of crisis such as the collapse of large financial institutions (Stuckler et al., 2012). Other effects of recession such as debt and house repossessions are likely to play a part in the mental health and distress of individuals which may be reflected in suicide rates. For example, home repossessions have been linked to a more than twofold increased rate of depressive symptoms and generalised anxiety disorder (GAD) (McLaughlin et al., 2012), both of which are risk factors for suicide.

As public sector and welfare spending is often cut during times of economic uncertainty, it is even more important to target preventive resources at those most vulnerable to the adverse effects of recession. This study aims to identify those groups most affected by the 2008 economic recession in England and Wales. Specifically we investigate:

1. Trends in suicide rates in England and Wales by gender, age-group and deprivation from 2001 to 2011 to identify changes in relation to a range of economic indicators.
2. Groups at increased risk of suicide following the 2008 economic recession by comparing the socio-demographic characteristics of suicide deaths in the three-years before the official start of the economic recession in the UK (April 2005–Mar 2008) with the subsequent three (April 2008–Mar 2011).

## 2 Methods

### 2.1 Data sources

#### 2.1.1 Suicide data for England and Wales

We obtained suicide mortality data from the Office for National Statistics. The data extract included all deaths by suicide for men and women in England and Wales occurring between 01 January 2001 and 31 December 2011 and which were registered by 31 December 2012. Classification of deaths for the study used the International Classification of Diseases, 10th edition (ICD-10) and included all deaths with a final underlying cause recorded as intentional self-harm (X60-X84), injury/poisoning of undetermined intent (Y10-Y34, except Y33.9) and sequelae of intentional self-harm, or injury/poisoning of undetermined intent (Y87.0, Y87.2), which follows conventional practice for government suicide statistics in the UK. In addition we included accidental poisoning (X40-X49), except X42 (accidental poisoning by and exposure to narcotics and psychodysleptics [hallucinogens], not elsewhere classified), and included accidental hanging (W76) to account for changes across the decade in potential mis-classification of suicides as a result of Coroner's growing use of narrative verdicts (Carroll et al., 2012, Hill and Cook, 2011). In sensitivity analysis we excluded accidental poisoning and accidental hanging cases.

#### 2.1.2 Population data

We used mid-year population estimates of the England and Wales population, which had recently been revised to take account of the most recent 2011 UK census, in order to calculate age-standardised suicide rates by gender and age-specific suicide rates by gender (ONSa, 2013, ONSb, 2013). Population estimates by index of multiple deprivation (IMD) decile, by 5-year age-groups (15–64 years) and by gender were provided by the Office for National Statistics.

#### 2.1.3 Economic data

(i) *Insolvencies and bankruptcy orders:* Data on the quarterly individual voluntary arrangements (IVAs) and bankruptcy orders (BOs) were obtained from the UK Insolvency Service (UKIS, 2012) and refer to new cases for adults in England and Wales aged 18 years and over. An IVA is an agreement between an individual and their creditors to pay off some or all of their debts over a period of time, whilst a BO is a court order used by individuals with little or no disposable income to write off unsecured debts.
(ii) *House repossessions:* Quarterly data on the number of mortgage claims leading to repossessions by county court bailiffs in England and Wales were obtained for the period 2001 to 2011 from the Ministry of Justice statistics (MoJ, 2013).
(iii) *Unemployment rate:* Data on UK quarterly unemployment rates and the percentage of the unemployed who were 'long term' unemployed (i.e. >12 months) for 16–64 year olds by gender were obtained from the ONS Labour Force Survey (ONSa, 2011). Unemployment rates were calculated using total economically active persons in this age-group as the denominator.
(iv) *Redundancy rate:* Quarterly redundancy rates for the period 2001 to 2011 by age-group and industry were available for the UK population of men and women aged 16 years and over from the ONS Labour Force Survey (ONSb, 2011).

Data for insolvencies, bankruptcy orders and house repossessions are for England and Wales only, whilst data on unemployment and redundancy rates are for the whole UK (England, Wales, Northern Ireland and Scotland). England and Wales combined account for 89% of the UK population.

#### 2.1.4 Calculating suicide rates

We determined annual and quarterly age-specific suicide rates per 100,000 in 16–64 year olds. Sex- and IMD quintile-specific suicide rates were age-standardised to the European Standard population. IMD specific suicide rates were calculated using the sample of 15–64 year olds because population data was not available excluding 15 year olds. Population estimates were not available by occupational status and therefore suicides rates by occupation could not be calculated. Suicide data used to calculate rates are based on the date of death and not the date of registration of death as commonly used in government statistics. In England and Wales, registration of suicide deaths cannot occur until after a Coroner's inquest has been conducted, which in the data used in this analysis occurred on average six-months after the death.

#### 2.1.5 Socio-demographic characteristics of suicides

*Age and gender:* we restricted our analysis to men and women aged 16–64 years, in keeping with official labour market statistics for working age adults.

*Occupation:* was recorded using the National Statistics Socio-Economic Classification (NS-SEC). The NS-SEC is an occupational-based classification based on the Standard Occupational Classification (SOC 2000) which codes occupation to unit groups but additionally incorporates employment relations and conditions of occupations. The five class version of NS-SEC was used, made up of five occupational classifications plus a never-worked/long-term unemployed group.

*Marital status:* was coded single, married, divorced, widowed, civil partnership, civil partnership widowed, civil partnership dissolved, unknown and not stated. For descriptive purposes we combined the following categories: a) civil partnership and married; b) civil partnership widowed and widowed; c) civil partnership dissolved and divorced; and d) unknown and not stated.

*Index of multiple deprivation decile:* The index of multiple deprivation (IMD) decile for England and Wales measured in 2010 for England and 2011 for Wales was supplied with the mortality data subset and defined as ‘1’ being most-deprived, ‘5’ intermediate deprived and ‘10’ being least-deprived. The English indices of deprivation identify the most deprived areas across the country and combine a number of indicators, chosen to cover a range of economic, social and housing issues, into one score for each small area in England (see Box 1). The Welsh index of multiple deprivation (WIMD) is not comparable with that of England and the number of suicides from Wales was too small to conduct country specific analysis using IMD score, hence analysis was restricted to England which comprised 93% of total deaths. IMD deciles were combined to create quintiles for the analysis of trends in suicide rates.

Box 1 **Construction of the English indices of multiple deprivation (IMD 2010).**

The English indices of deprivation combine information in 38 separate indicators into seven distinct domains: income; employment; health and disability; education, skills and training; barriers to housing and services; crime; and living environment (McLennan et al., 2011). These domains are combined, using appropriate weights, and a score is calculated for every Lower layer Super Output Area (LSOA) in England (LSOA *n* = 34,753 in England and Wales). The IMD can be used to rank every LSOA according to its relative level of deprivation. For this study LSOAs have been ranked and transformed into deciles and quintiles according to IMD score.

#### 2.1.6 Statistical analyses

Analyses were carried out using Stata version 12.1 (StataCorp, 2011) unless otherwise stated. We used Joinpoint regression analysis software (Joinpoint Version 4.0.4) to identify changes in trends in quarterly suicide rates across the study period 2001–2011. We assumed Poisson distributed variation in suicide rates over time, and uncorrelated errors from the models. We chose the grid-search method to identify the change points and the software set to a default maximum number of joinpoints which in this case was five. The final number of change points is determined by comparing the best model with each number of change points using permutation tests, with a Bonferroni correction to account for multiple hypothesis testing (Kim et al., 2000). The results are presented as straight lines connected at change points, on a log scale, and these trends are characterised with a quarterly percentage change (QPC) between successive change points. All estimates are presented with 95% confidence intervals.

We tested whether the characteristics of suicides before and after the onset of the recession differed by comparing the distribution of suicides by age, marital status, occupational class and deprivation decile in the 3-years up to March 2008 versus the 3-years from April 2008 onwards using Pearson's chi-square statistic.

## 3 Results

### 3.1 Trends in economic indicators in the UK, 2001–2011

**Fig. 1:**
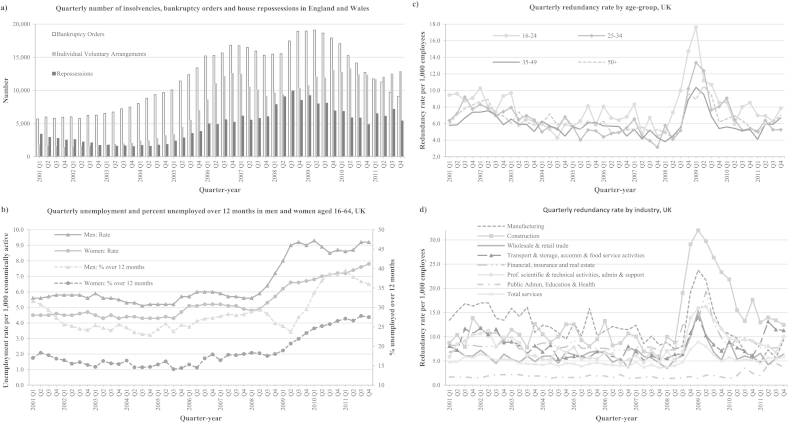
Quarterly economic indicators for the UK 2001–2011.

Fig. 1 shows trends in several economic indicators by quarter-year from 2001 to 2011. Indicators of debt began increasing well before the onset of the economic recession in 2008 (Fig. 1a). House repossessions increased steadily from 2004 to a peak in 2008, whilst Bankruptcy Orders (BOs) and Individual Voluntary Arrangements (IVAs) increased from 2003 and both reached an initial peak in 2007, then after a slight dip increased to a second peak in 2009–2010. The decline seen in BOs from mid-2009 may reflect the introduction of debt relief orders (DROs) around this time.

Quarterly unemployment rates in working age men and women (Fig. 1b) were at their decade lowest for the period 2001 to 2005 after which unemployment rose slightly then plateaued until the sharp rises in unemployment seen from Q2 2008 in both men and women. Following the sharp rises in unemployment, rates in men fell slightly in 2010 and then plateaued whilst in women rates continued to increase to the end of 2011. The percentage of men and women unemployed long term followed a similar, albeit lagged, pattern. Quarterly redundancy rates across all age-groups (Fig. 1c) and industry sectors (Fig. 1d) had been in decline from the end of 2001 to the first quarter of 2008 after which they rose sharply in the second quarter of 2008 then sharply fell in 2009. The highest rises in redundancies were seen in the younger age-groups (16–34 years) where rates rose four-fold from a mean rate of around 3.8 per 1000 to 15.5 per 1000 (Fig. 1c). The highest rises in industry-specific redundancy rates occurred in the construction and manufacturing sectors with peaks in 2009 of 32.0 per 1000 employees and 23.8 per 1000 respectively. In 2008, construction accounted for about 8% of the total workforce jobs in the UK and manufacturing about 10% of the workforce. The third highest rise in redundancies was seen in the financial sector, which makes up around 20% of the workforce in the UK, although their peak was slightly later, in the second quarter of 2009. In the public administration, health and education sector, which make-up around 24% of the UK workforce, there was little increase in redundancies during the 2008 to 2009 period but a small increase during 2010.

### 3.2 Trends in suicide rates in England and Wales, 2001–2011

A summary of the quarterly percent change (QPC) in suicide rates and joinpoints (JP) for the modelled trends in age-standardised suicide rates, age-specific suicide rates for men and women in England and Wales and age-standardised suicide rates by IMD (Indices of Multiple Deprivation) quintile for men and women in England, 2001–2011 can be found in Table 1a and Table 1b respectively.

**Table 1a:** Summary of quarterly percent change (QPC^a^) in suicide rates and joinpoints (JP^b^) for modelled trends in age-standardised suicide rates and age-specific suicide rates for men in England and Wales and age-standardised suicide rates by IMD (Indices of Multiple Deprivation) quintile for men in England, 2001-2011.

	Segment 1		JP 1	(95% CI)	Segment 2		JP 2	(95% CI)	Segment 3		JP 3	(95% CI)	Segment 4		JP 4	(95% CI)	Segment 5	
	QPC	(95% CI)			QPC	(95% CI)			QPC	(95% CI)			QPC	(95% CI)			QPC	(95% CI)
Male																		
ASR	−0.6^c^	(−0.8, −0.3)	Q2 2006	(Q3 2001, Q4 2007)	1.1	(-0.4, 2.6)	Q2 2008	(Q2 2004, Q1 2010)	−0.2	(-0.8, 0.3)								
Age-group																		
16–24	−1.9^c^	(−2.7, −1.1)	Q2 2006	(Q3 2004, Q3 2007)	2.7	(-1.6, 7.2)	Q2 2008	(Q3 2006, Q4 2009)	−1.9^c^	(-3.4, −0.5)								
25–34	−0.3	(−1.1, 0.5)	Q3 2004	(Q3 2001, Q3 2005)	−5.4^c^	(-9.5, −1.2)	Q1 2006	(Q1 2005, Q4 2006)	8.4	(-10.9, 31.8)	Q4 2006	(Q2 2006, Q2 2009)	−0.9^c^	(-1.5, −0.4)				
35–44	2.6	(−0.8, 6.2)	Q2 2002	(Q3 2001, Q4 2007)	−0.3	(-0.7, 0.1)	Q3 2007	(Q2 2004, Q4 2008)	5.9	(-8.3, 22.3)	Q2 2008	(Q3 2007, Q3 2010)	−2.1^c^	(-4.0, −0.2)	Q2 2010	(Q4 2008, Q2 2011)	2.6^c^	(0.1, 5.1)
45–54	0.5^c^	(0.3, 0.6)																
55–64	0.4^c^	(0.1, 0.6)																
IMD																		
1st quintile	−0.6^c^	(−0.8, −0.5)																
2nd quintile	−0.4^c^	(−0.6, −0.2)																
3rd quintile	−0.3^c^	(−0.5, 0.0)																
4th quintile	−0.3	(−0.5, 0.0)																
5th quintile	0.3	(0.0, 0.5)																

a QPC coefficients are the quarterly percent change in suicide rates between the specified join points. Negative coefficients indicate downward trends. Positive coefficients indicate upward trends.

b JPs indicate the quarter-years when a change in trends in suicide rates is estimated.

c The QPC is significantly different from zero at alpha = 0.05.

**Table 1b:** Summary of quarterly percent change (QPC^a^) in suicide rates and joinpoints (JP^b^) for modelled trends in age-standardised suicide rates and age-specific suicide rates for women in England and Wales and age-standardised suicide rates by IMD (Indices of Multiple Deprivation) quintile for women in England, 2001-2011.

	Segment 1		JP 1	(95% CI)
	QPC	(95% CI)		
Female				
ASR	−0.1	(−0.3, 0.0)		
Age-group				
16–24	−0.7^c^	(−1.2, −0.2)		
25–34	−0.4^c^	(−0.8, −0.1)		
35–44	−0.1	(−0.4, 0.2)		
45–54	0.3^c^	(0.0, 0.5)		
55–64	−0.1	(−0.4, 0.2)		
IMD				
1st quintile	−0.7^c^	(−1.1, −0.4)		
2nd quintile	−0.7^c^	(−1.0, −0.3)		
3rd quintile	−0.4	(−0.7, 0.0)		
4th quintile	−0.6^c^	(−0.9, −0.2)		
5th quintile	0.0	(−0.4, 0.4)		

a QPC coefficients are the quarterly percent change in suicide rates between the specified join points. Negative coefficients indicate downward trends. Positive coefficients indicate upward trends.

b JPs indicate the quarter-years when a change in trends in suicide rates is estimated.

c The QPC is significantly different from zero at alpha = 0.05.

**Fig. 2:**
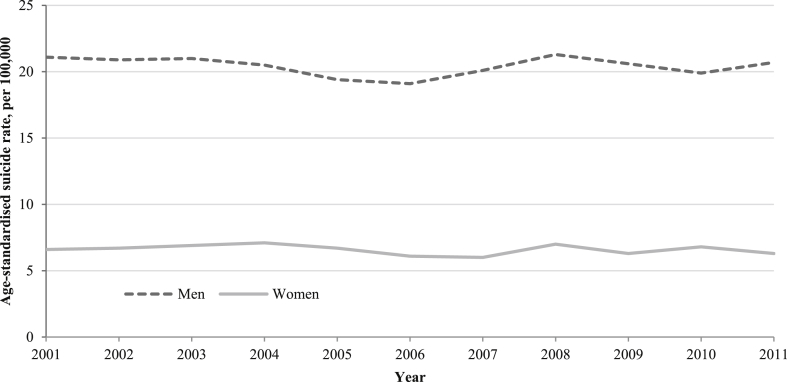
Trends in annual age-standardised suicide rates in men and women aged 16–64 years old in England and Wales, 2001–2011.

Annual age-standardised suicide rates for men and women aged 16–64 years in England and Wales (Fig. 2) appeared relatively stable between 2001 and 2011. In men suicide rates declined from 21.1 per 100,000 in 2001 to their decade lowest in 2006 at 19.1 per 100,000 then began to rise to 20.1 per 100,000 in 2007 and reached a peak in 2008 at 21.3 per 100,000. In women the highest suicide rates were in 2004 and 2008 at 7.1 and 7.0 per 100,000 respectively, whilst the lowest rate was in 2007 at 6.0 per 100,000.

The modelled trends in quarterly age-standardised suicide rates for men show evidence of a downward trend for the period up to Q2 2006 (QPC −0.6, 95% CI −0.8 to −0.3), reversing to an upward trend for the period to Q2 2008 (QPC 1.1, 95% CI −0.4, 2.6) and returning to a slight downward trend to the end of 2011 (QPC −0.2, 95% CI −0.8, 0.3), although statistical evidence for these latter two trends is weak (Table 1a). The modelled trends appear to match those seen in the observed annual age-standardised trends in men (Fig. 2). Some caution is required in interpreting the timing of these changes in trends as we see from the 95% CI that the initial joinpoint may have occurred from Q3 2001 to Q4 2007, whilst the second joinpoint may have occurred from Q2 2004 to Q1 2010. In women the model-based estimates show a consistent, small downward trend in quarterly age-standardised suicide rates (QPC -0.1, 95% CI -0.3 to 0.0) across the study period from Q1 2001 to Q4 2011 (Table 1b).

**Fig. 3:**
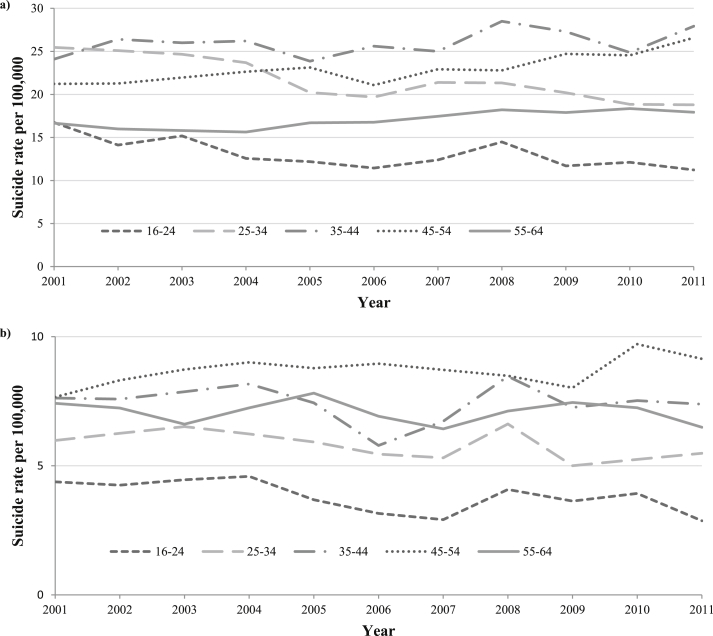
Trends in age-specific suicide rates in men (a) and women (b) aged 16–64 years in England and Wales, 2001–2011 (note scale difference: for males *y*-axis ranges from 0–30 per 100,000; for females *y*-axis ranges from 0–10 per 100,000).

Fig. 3 shows the trends in annual age-specific suicide rates in working age men (Fig. 3a) and women (Fig. 3b). Suicide rates in younger men (16–34) were at their highest point in 2001 and declined to their lowest point in 2011. Conversely, for older men (35–64), rates increased between 2001 and 2011.

Increases in suicide rates around the time of the onset of the recession (in 2008) were seen in 16–24 and 35–44 year old men. In 16–24 year old men rates rose from 11.5 per 100,000 in 2006 to 14.5 per 100,000 in 2008 (a 26% rise), whilst in the 35–44 year old men rates rose from 25.0 per 100,000 in 2007 to 28.5 per 100,000 in 2008 a 14% rise over one year. Suicide rates in men aged 25–34 years appeared to follow a downward trend to 2006 at which point they changed to an upward trend to the end of 2011. Suicide rates in 45–64 year old men increased steadily throughout the 10 year period. In women there were peaks in suicide rates in 16–24, 25–34 and 35–44 year olds in 2008 with 28%, 20% and 47% rises compared to 2006, there was a similar rise in 45–54 year olds in 2010. For the modelled trends in age-specific suicide rates in men (Table 1a), there was evidence of a pause in the downward trend in rates for 16–24 year olds starting around Q2 2006 (95% CI Q3 2004, Q3 2007) and in 25–34 year olds starting around Q1 2006 (95% CI Q1 2005, Q4 2006). These changes in trends in suicide rates in 16–34 year olds coincide with increases in indicators of financial strain (Fig. 1a). In 35–44 year old men modelled suicide rates appeared to rise until Q2 2008 (Q3 2007, Q3 2010) when there was evidence of a decline in rates to Q2 2010 (95% CI Q4 2008, Q2 2011) followed by an increase in rates (QPC 2.6 95% CI 0.1, 5.1). Although it is not clear because of the wide confidence intervals around these change points in trends it may be that the increase in suicide rates at the end of the decade in 35–44 year old men coincided with peaks in unemployment, redundancy and long-term unemployment rates. There was no evidence for any change in the modelled trends (joinpoints) in age-specific suicide rates in women (Table 1b).

**Fig. 4:**
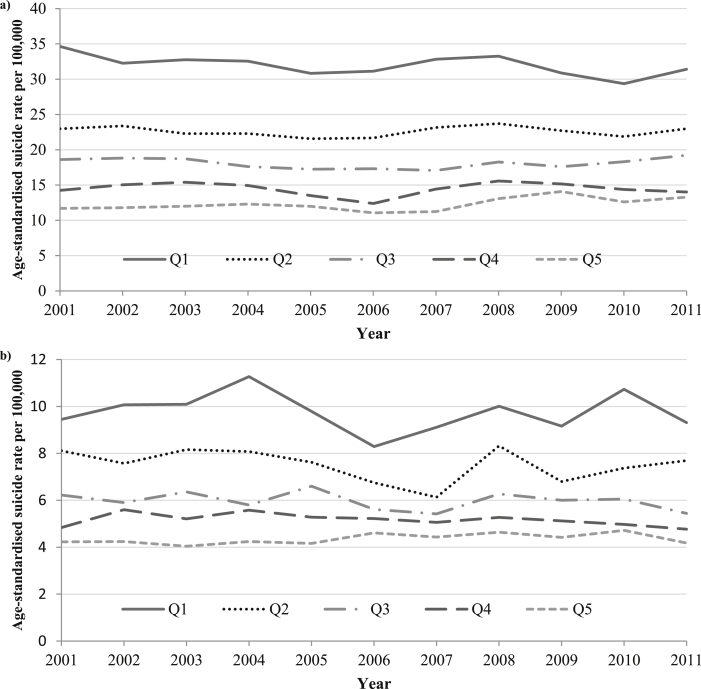
Trends in annual age-standardised suicide rates by IMD quintile (1st = most deprived to 5th = least deprived) in men (a) and women (b) aged 15–64 years in England, 2001–2011 (note scale difference: for males *y*-axis ranges from 0–40 per 100,000; for females *y*-axis ranges from 0–12 per 100,000).

Fig. 4 shows trends in the annual age-standardised suicide rates by Indices of Multiple Deprivation (IMD) score quintiles in men (Fig. 4a) and women (Fig. 4b) aged 15–64 in England. The most striking feature of this figure is the three fold difference in suicide rates between the 20% most-deprived (1st quintile) and 20% least-deprived (5th quintile) areas throughout the period. Suicide rates amongst men living in the least-deprived areas (5th quintile) rose from 11.2 per 100,000 in 2007 to 13.3 per 100,000 by 2011. Suicide rates amongst men living in the most-deprived 20% of areas (1st quintile) began the decade at 34.6 per 100,000 but had declined to 31.4 per 100,000 by 2011, with an observed small rise between 2005 and 2008. Annual age-standardised suicide rates in the least-deprived 20% of women in England (Fig. 4b) were relatively stable across the decade; they began (2001) and ended (2011) the decade at 4.2 per 100,000. Suicide rates in the most-deprived 20% of women in England showed evidence of a downward trend across the period.

The modelled trends in quarterly suicide rates by IMD quintile for men found no statistical evidence of a change in trends by area level deprivation quintile (Table 1a). There was evidence of a downward trend in suicide rates across the decade for men living in the most-deprived to medium-deprived areas, and largely stable suicide rates for men living in the least-deprived areas. The only increase in suicide rates across the decade was seen in the least deprived areas (QPC 0.3 95% CI 0.0, 0.5). There was evidence of a downward trend in suicide rates across the decade for women living in the 1st and 2nd most deprived areas and the 2nd least deprived areas (Table 1b).

### 3.3 Characteristics of pre- and post-recession suicide deaths

Table 2 shows the frequency and distribution of suicide deaths by characteristic in working age (16–64 years) men and women in England and Wales occurring in the 3-year period before the 2008 economic recession (April 2005–March 2008) and the 3-year period during/after the onset of the economic recession (April 2008–March 2011). There was evidence of a difference in the distribution of suicides by age-group, occupation and IMD decile in the recession period *vs* the pre-recession years in men, but not in women. A higher proportion of suicides occurred amongst men in the older age categories (>45 years) during/after the recession. There was no evidence of a difference in the marital status of suicide cases pre- and post-recession.

**Table 2:** Comparing the distribution of characteristics of male and female suicide deaths age 16–64 years old in England and Wales in the three years before (April 2005–Mar 2008) and three years during/after (April 2008–Mar 2011) the 2008 economic recession.

Characteristic	Male (*n* = 21,469)				Female (*n* = 6,979)			
	Apr 2005–Mar 08		Apr 2008–Mar 11		Apr 2005–Mar 08		Apr 2008–Mar 11	
	*n*	%	*n*	%	*n*	%	*n*	%
Age-group								
16–24	1153	11.2	1255	11.3	317	9.4	366	10.2
25–34	2181	21.1	2187	19.7	621	18.4	610	16.9
35–44	3095	29.9	3206	28.8	829	24.6	927	25.7
45–54	2320	22.4	2712	24.4	919	27.2	980	27.2
55–64	1590	15.4	1770	15.9	690	20.4	720	20.0
*X*^*2*^*p-value*				0.002				*0.367*
Marital Status								
Single	4852	51.4	5170	52.9	1191	37.7	1242	39.2
Married	3012	31.9	3031	31.0	1110	35.2	1118	35.3
Divorced	1426	15.1	1404	14.4	696	22.1	660	20.9
Widowed	157	1.7	174	1.8	159	5.0	145	4.6
*X*^*2*^*p-value*^a^				0.141				*0.443*
NS-SEC occupations								
Higher management, administrative and professional	1661	20.9	1833	21.6	544	26.2	645	28.3
Intermediate	639	8.0	635	7.5	404	19.5	432	18.9
Small employers and own account workers	1032	13.0	1163	13.7	61	2.9	49	2.2
Lower supervisory and technical	907	11.4	939	11.1	34	1.6	42	1.8
Semi-routine and routine	3366	42.3	3510	41.3	887	42.8	935	41.0
Never worked and long-term unemployed	93	1.2	83	1.0	34	1.6	29	1.3
Full-time student	251	3.2	337	4.0	111	5.4	151	6.6
*X*^*2*^*p-value*^b^				0.024				*0.146*
IMD Decile^c^								
Most-deprived 1st	1867	18.1	1904	17.1	541	16.0	595	16.5
2nd	1424	13.8	1463	13.1	389	11.5	446	12.4
3rd	1248	12.1	1410	12.7	399	11.8	456	12.7
4th	1136	11.0	1188	10.7	349	10.3	349	9.7
5th	1037	10.0	1031	9.3	350	10.4	349	9.7
6th	899	8.7	983	8.8	295	8.7	338	*9.4*
7th	764	7.4	880	7.9	278	8.2	308	8.6
8th	729	7.1	798	7.2	281	8.3	282	7.8
9th	671	6.5	797	7.2	263	7.8	238	6.6
Least-deprived 10th	564	5.5	676	6.1	231	6.8	242	6.7
*X*^*2*^*p-value*				0.027				0.451

a *X*^*2*^ test for marital status analysis excluding ‘not stated’ category.

b *X*^*2*^ test for ns-sec analysis excluding ‘not stated’ category.

c Data for England only.

There was no consistent pattern of change in the distribution of suicides across categories from high to low socioeconomic group although there was a slight rise in the proportion of suicides in the higher management and professional group, small employer/self-employed and in fulltime students and a fall in the semi-routine/routine groups during/after the 2008 economic recession. Coinciding with the higher proportion of suicides in higher socio-economic groups there was a rise in the proportion of suicides amongst men living in the least deprived areas (decile 6th–10th), reflecting a larger increase in suicides in the least compared to most deprived areas. There was little evidence of differences in the socio-demographic characteristics of female suicides pre- and post-recession. Following examination of the economic data, which suggested an earlier experience of economic stress, we tested different cut-points for pre- and post-recession periods. Changing the cut-points by plus or minus one-year did not affect our results.

### 3.4 Sensitivity analysis

The analysis was repeated using the sensitivity sample restricted to only suicide and undetermined deaths. Using this sample, trends in age-standardised suicide rates and age-specific suicide rates in men and women followed similar patterns of change across the decade as in the main sample. However, as expected, in general suicide rates in the sensitivity sample were lower with this gap widening as the decade progressed (Appendix 2, Appendix 3 and Appendix 4). Significant downward trends in the modelled annual age-standardised suicide rates were found in men and women in the sensitivity sample and there was no evidence of any change in trends across the decade for either gender. Pre- and post-recession characteristics comparison showed an almost identical change in distributions as seen in the primary sample (see Appendix 5).

## 4 Discussion

### 4.1 Main findings

Suicide rates in men began the decade in decline with evidence that this downward trend halted around 2006. The halt in the downward trend in suicide rates in men around 2006 was most evident in men aged 16–34 year olds, whilst for men aged 35–44 years it appeared suicide rates were generally on the rise until around 2008 (95% CI Q3 2007, Q3 2010), after which followed a short period of reducing rates and then a return to an upward trend from 2010 (95% CI Q4 2008, Q2 2011). Suicide rates in 45–64 year old males rose continuously from 2001 to 2011. There was no statistical evidence of a similar rise in women, although an observed short-term peak in suicide rates was seen in 2008 in 16–44 year olds.

Whilst the economic recession began officially in the second quarter of 2008, trends in some of the economic measures we investigated (bankruptcy/house repossessions) indicated that financial pressures on individuals and households predated 2008 by several years and may have contributed to the halt in the downward trend in male suicides in Q2 in 2006 (CI Q3 2001-Q4 2007). In addition rises in redundancies from early 2008, which were likely to have been preceded by concerns about possible job-loss, and then unemployment, compounded by difficulties obtaining new employment, all may have contributed to the reversal in the suicide rate trend from downward to upward in men aged 35–44 years old around 2010 (95% CI Q4 2008, Q2 2011).

Suicide rates were considerably higher amongst those living in the more deprived areas of England, although there was no evidence that rates in these areas increased more during the 2008 economic recession than those in more affluent areas. Indeed suicide rates decreased throughout the study period in males living in the more deprived areas whereas they were relatively stable in the least-deprived areas. There was no evidence of change in the distribution of suicides according to marital status. There was some evidence of a rise in the proportion of suicides amongst males in the period during/after the 2008 economic recession whose occupation was recorded as student, higher management/professional or small employers and own account workers, with reductions in the other occupational groups. There was a higher proportion of suicides found amongst men in older age categories (>45 years) in the during/after recession period although this may reflect the year on year rises in suicide rates in this age group, rather than an effect of the recession.

### 4.2 Strengths and limitations

This is the first assessment of age- and sex-specific trends in suicide in the years before and after the economic recession of 2008 in relation to a variety of indicators of recession effects. We used date of death rather than date of registration of death to enable us to investigate trends and timing more precisely. Furthermore we took account of recent changes in the coding of possible suicide deaths in England and Wales (Carroll et al., 2012, Gunnell et al., 2012) by including accidental hanging and accidental poisoning deaths in our primary analysis. In a sensitivity analysis we excluded accidental hangings and poisonings and found that overall patterns in trends in suicide rates did not differ between samples, however the sensitivity sample showed an overall downward trend in annual age-standardised suicide rates in men and women in contrast to the primary sample where rates remained equal or higher than at the beginning of the decade. This suggests a detrimental impact of recent changes in coroners choice of verdict for studies on analysis of trends in suicide.

Data are available for too few years before and after the 2008 economic recession, and are too highly inter-correlated, to enable us to undertake a meaningful multivariable time series analysis. Nevertheless, our primary aim was to determine whether suicide rates rose around the time of the onset of the economic recession, rather than identify independent effects of particular economic indicators. Furthermore, as this is an ecological study, we cannot determine whether the increase in suicide was due to increased numbers of suicides amongst people who lost their jobs, were made homeless or suffered other stresses related to the 2008 economic recession.

### 4.3 Findings in relation to previous literature

A large body of literature has documented the adverse effect of periods of economic recession on suicide rates (Chang et al., 2009, Chang et al., 2013, Khang et al., 2005, Stuckler et al., 2009, Stuckler et al., 2012, Swinscow, 1951). Our findings are in keeping with Barr's earlier analyses of data for England, although as these authors used date of death registration rather than date of death in their analysis [Personal Communication, Barr B,] they may have underestimated the extent of the pre-2008 rise in suicide (Barr et al., 2012). In their analysis of trends in suicide across English regions in relation to changes in unemployment, Saurina et al. found mixed evidence that rises in unemployment were associated with increases in suicide (Saurina et al., 2013). We found that the rise in suicide around the time of the 2008 economic recession in England and Wales was largely restricted to 35–44 year old males; whereas recent analysis of World Health Organisation data on suicides before and after the economic recession in 54 countries largely attributed rises in the number of suicides in European men to the 15–24 year old age group (Chang et al., 2013). In our study we found evidence that in this younger age-group of 16–24 year olds a halt in the downward trend in suicide rates is likely to have occurred before the 2008 economic recession.

Whilst rises in unemployment are commonly used as an indicator of economic recession, previous research has shown that periods of recession are characterised by a range of other stresses as well as job losses. These include financial and relationship strain, cuts in health services and the effects of austerity measures such as welfare reforms on already vulnerable people (Hawton and Haw, 2013, Kentikelenis et al., 2011, Stuckler et al., 2009). In Barr's analysis of suicide in England and Wales before and after 2008, changes in unemployment accounted for approximately 40% of the rise in suicides (Barr et al., 2012). In analyses of the 1998 East Asian financial crisis, whilst rises in levels of unemployment appeared to explain some of the increases in suicide in Hong Kong and South Korea, they did not appear to influence the impact of the recession on suicide in Japan (Chang et al., 2009). Most significantly, Stuckler and colleagues in their analysis of the effects on mortality of economic recession in Europe, found that differences between countries in the rises in suicide that accompanied recessions were influenced by differences between nations in their spending on active labour market programmes (Stuckler et al., 2009). Those countries with higher levels of spending experienced smaller rises in suicide as unemployment rose. Improvements in welfare provision in England and Wales since the great depression of the 1920s/1930s might also explain why the impact of that recession on suicides (Swinscow, 1951) was far greater than that of the 2008 economic recession, although other factors, including improved recognition and treatment of mental illness may also have contributed.

Studies of coroners' records have shown that financial strain, job loss and loss of home are common contributors to suicide. In a recent analysis of suicides in Wales, debt was mentioned as a relevant factor in 11% of male suicides (Scourfield et al., 2012). Stack and Wasserman (Stack and Wasserman, 2007) in their study of 675 suicides in an urban county of the USA found economic strain was present in 9% of cases – strains included loss of a home (10 cases), and job loss (33 cases). Recent epidemiological research from the USA found an association between home foreclosures and suicide rates between 2005 and 2010, particularly in middle-aged men (46–64 years old) (Houle and Light, 2014), similarly a growing number of studies are documenting the adverse impact on mental health of unemployment and financial difficulties (Eliason and Storrie, 2009, Jackson and Warr, 1984, McKee-Ryan et al., 2005, Skapinakis et al., 2006).

### 4.4 Conclusions and policy implications

Much of the research to date on suicide and recession has highlighted rises in suicide associated with sharp rises in unemployment. In our study we show that a downward trend in suicide rates in young men aged 16–34 years old halted for a period before/during the 2008 economic recession. For the 25–34 year olds this halt in decreasing suicide rates may be partly explained by pre-recession increases in indicators of financial strain such as house repossession and insolvencies, whilst in the 16–24 year olds just entering the labour market rising rates of unemployment from 2004 may have contributed (ONSc, 2013). In men aged 35–44 years there was a reversal from a downward to upward trend in suicide rates estimated to be between the end of 2008 and the middle of 2011 which corresponds to a number of economic factors such as peaks in redundancies, unemployment and long-term unemployment as well as rises in individual insolvencies. These rises seen in indicators of financial stress and hardship such as house repossessions and household debt or indeed other unmeasured factors such as anticipated job loss may also have contributed to the changes in trends in suicide rates. Our analysis supports the hypothesis that the association between economic recession and suicide rates in men is not straight-forward and, as with other health indicators, may commence with the anticipation of unemployment rather than unemployment per se (Perlman and Bobak, 2009, Stuckler et al., 2012). We found evidence that the risk of suicide in some groups, most notably young males, may have been more affected than other age/sex groups, but we found no evidence of an increased risk amongst people living in more deprived areas. This may suggest that welfare safety nets for men and women in lower socio-economic groups have been effective in protecting these groups from the effects of the recession. Alternatively, the apparent lack of an effect of the 2008 economic recession on the most-deprived and those in the lower socio-economic groups may reflect the relative lack of change in their economic situation over the period of our analysis as they are more likely to have been living in social/local authority rented accommodation and to have insecure employment pre-recession. However, with the UK Government's introduction of the Welfare Reform Act 2012, increased pressure is being placed on those supported by social security with measures such as benefit caps and restrictions on housing benefit (“bedroom tax”). In addition, cuts in local government spending and consequent public sector job losses have occurred since the study time period so longer term analysis is needed to understand the full consequences of the recession and policy responses to it. Our study suggests that interventions for suicide prevention around economic recession targeted at those struggling with debt or at threat of redundancy might be more effective than those solely targeted at the unemployed.
