# Supplementary material for: Suicide and the 2008 economic recession: Who is most at risk? Trends in suicide rates in England and Wales 2001–2011
Source: Soc Sci Med. 2014 Sep;117:76–85. doi: 10.1016/j.socscimed.2014.07.024 (PMC4151136; doi:10.1016/j.socscimed.2014.07.024)
Supplement: Supplementary file 1 [file mmc1.docx]

**Appendix. 1.** Quarterly UK GDP 2001-2011: year on year growth and recession start
