# Supplementary material for: Suicide and the 2008 economic recession: Who is most at risk? Trends in suicide rates in England and Wales 2001–2011
Source: Soc Sci Med. 2014 Sep;117:76–85. doi: 10.1016/j.socscimed.2014.07.024 (PMC4151136; doi:10.1016/j.socscimed.2014.07.024)
Supplement: Supplementary file 3 [file mmc3.docx]

**Appendix. 3.** Trends in age-specific suicide rates in men (a) and women (b) aged 16-64 years in England and Wales, 2001-2011, suicides and undetermined deaths only
