# Supplementary material for: Suicide and the 2008 economic recession: Who is most at risk? Trends in suicide rates in England and Wales 2001–2011
Source: Soc Sci Med. 2014 Sep;117:76–85. doi: 10.1016/j.socscimed.2014.07.024 (PMC4151136; doi:10.1016/j.socscimed.2014.07.024)
Supplement: Supplementary file 5 [file mmc5.docx]

**Appendix. 5.** Comparing the distribution of characteristics of male and female suicide deaths age 16-64 years old in England and Wales in the three years before (April 2005-Mar 2008 ) and three years during/after (April 2008-Mar 2011) the 2008 economic recession, suicides and undetermined deaths only

| **Characteristic** | **Male**  (n=17,777) | | | | **Female**  (n=5,344) | | | |
| --- | --- | --- | --- | --- | --- | --- | --- | --- |
|  | **Apr 2005-Mar 08** | | **Apr 2008-Mar 11** | | **Apr 2005-Mar 08** | | **Apr 2008-Mar 11** | |
|  | *n* | % | *n* | % | *n* | % | *n* | % |
| Age-group |  |  |  |  |  |  |  |  |
| 16-24 | *953* | 10.9 | *1,007* | 11.2 | *245* | 9.2 | *284* | 10.6 |
| 25-34 | *1,806* | 20.6 | *1,708* | 18.9 | *493* | 18.5 | *458* | 17.1 |
| 35-44 | *2,622* | 29.9 | *2,558* | 28.4 | *634* | 23.8 | *674* | 25.2 |
| 45-54 | *1,966* | 22.4 | *2,232* | 24.8 | *736* | 27.6 | *725* | 27.1 |
| 55-64 | *1,414* | 16.1 | *1,511* | 16.8 | *558* | 20.9 | *537* | 20.1 |
| *X^2^ p-value* |  |  |  | *<0.001* |  |  |  | *0.211* |
| Marital Status |  |  |  |  |  |  |  |  |
| Single | *4,006* | 49.8 | *4,061* | 50.9 | *938* | 37.6 | *925* | 39.3 |
| Married | *2,681* | 33.3 | *2,632* | 33.0 | *901* | 36.1 | *849* | 36.1 |
| Divorced | *1,213* | 15.1 | *1,134* | 14.2 | *537* | 21.5 | *480* | 20.4 |
| Widowed | *142* | 1.8 | *147* | 1.8 | *122* | 4.9 | *98* | 4.2 |
| *X^2^ p-value*^a^ |  |  |  | *0.350* |  |  |  | *0.383* |
| NS-SEC occupations |  |  |  |  |  |  |  |  |
| Higher management, administrative and professional | *1,486* | 21.5 | *1,581* | 22.3 | *457* | 27.2 | *522* | 29.5 |
| Intermediate | *569* | 8.2 | *548* | 7.7 | *338* | 20.1 | *336* | 19.0 |
| Small employers and own account workers | *895* | 12.9 | *976* | 13.7 | *48* | 2.9 | *41* | 2.3 |
| Lower supervisory and technical | *787* | 11.4 | *801* | 11.3 | *26* | 1.6 | *29* | 1.6 |
| Semi-routine and routine | *2,887* | 41.8 | *2,857* | 40.2 | *701* | 41.7 | *704* | 39.8 |
| Never worked and long-term unemployed | *68* | 1.0 | *60* | 0.8 | *24* | 1.4 | *21* | 1.2 |
| Full-time student | *223* | 3.2 | *283* | 4.0 | *87* | 5.2 | *116* | 6.6 |
| *X^2^ p-value*^b^ |  |  |  | *0.059* |  |  |  | *0.308* |
| IMD Decile^c^ |  |  |  |  |  |  |  |  |
| Most-deprived 1^st^ | *1,503* | 17.2 | *1,411* | 15.7 | *380* | 14.3 | *383* | 14.3 |
| 2^nd^ | *1,202* | 13.7 | *1,129* | 12.5 | *302* | 11.3 | *316* | 11.8 |
| 3^rd^ | *1,040* | 11.9 | *1,098* | 12.2 | *327* | 12.3 | *316* | 11.8 |
| 4^th^ | *948* | 10.8 | *946* | 10.5 | *286* | 10.7 | *259* | 9.7 |
| 5^th^ | *888* | 10.1 | *874* | 9.7 | *259* | 9.7 | *282* | 10.5 |
| *6^th^* | *775* | *8.9* | *832* | *9.2* | *247* | *9.3* | *270* | *10.1* |
| 7^th^ | *675* | 7.7 | *753* | 8.4 | *217* | 8.1 | *235* | 8.8 |
| 8^th^ | *641* | 7.3 | *679* | 7.5 | *236* | 8.9 | *226* | 8.4 |
| 9^th^ | *583* | 6.7 | *688* | 7.6 | *208* | 7.8 | *189* | 7.1 |
| Least-deprived 10^th^ | *506* | 5.8 | *606* | 6.7 | *204* | 7.7 | *202* | 7.5 |
| *X^2^ p-value* |  |  |  | 0.001 |  |  |  | 0.771 |

^a^ *X^2^* test for marital status analysis excluding ‘not stated’ category

^b^ *X^2^* test for ns-sec analysis excluding ‘not stated’ category

^c^ data for England only
